# Alterations in Regulatory T Cells Induced by Specific Oligosaccharides Improve Vaccine Responsiveness in Mice

**DOI:** 10.1371/journal.pone.0075148

**Published:** 2013-09-20

**Authors:** Marcel A. Schijf, JoAnn Kerperien, Jacqueline Bastiaans, Kirsten Szklany, Jenny Meerding, Gerard Hofman, Louis Boon, Femke van Wijk, Johan Garssen, Belinda van’t Land

**Affiliations:** 1 Department of Pediatric Immunology, Wilhelmina Children’s Hospital, University Medical Center Utrecht, Utrecht, The Netherlands; 2 Department of Immunology, Nutricia Research, Utrecht, The Netherlands; 3 Utrecht Institute for Pharmaceutical Sciences (UIPS), Utrecht University, Utrecht, The Netherlands; 4 Bioceros, Utrecht, The Netherlands; National Institute of Allergy and Infectious Diseases, United States of America

## Abstract

Prophylactic vaccinations are generally performed to protect naïve individuals with or without suppressed immune responsiveness. In a mouse model for Influenza vaccinations the specific alterations of CD4^+^CD25^+^Foxp3^+^ regulatory T-cells (Tregs) in the immune modulation induced by orally supplied oligosaccharides containing scGOS/lcFOS/pAOS was assessed. This dietary intervention increased vaccine specific DTH responses. In addition, a significant increased percentage of T-bet^+^ (Th1) activated CD69^+^CD4^+^ T cells (p<0.001) and reduced percentage of Gata-3^+^ (Th2) activated CD69^+^CD4^+^T cells (p<0.001) was detected in the mesenteric lymph nodes (MLN) of mice receiving scGOS/lcFOS/pAOS compared to control mice. Although no difference in the number or percentage of Tregs (CD4^+^Foxp3^+^) could be determined after scGOS/lcFOS/pAOS intervention, the percentage of CXCR3 ^+^ /T-bet^+^ (Th1-Tregs) was significantly reduced (p<0.05) in mice receiving scGOS/lcFOS/pAOS as compared to mice receiving placebo diets. Moreover, although no absolute difference in suppressive capacity could be detected, an alteration in cytokine profile suggests a regulatory T cell shift towards a reducing Th1 suppression profile, supporting an improved vaccination response.

**In conclusion:**

These data are indicative for improved vaccine responsiveness due to reduced Th1 suppressive capacity in the Treg population of mice fed the oligosaccharide specific diet, showing compartmentalization within the Treg population. The modulation of Tregs to control immune responses provides an additional arm of intervention using alternative strategies possibly leading to the development of improved vaccines.

## Introduction

The induction of proper immune responsiveness to vaccinations shortly after birth or in immune compromised individuals is challenging. The highly protective environment and need to avoid immunological interactions of the fetus against the mother seem to be the main reason for this “physiological” immaturity of the immune system in newborn infants. Regulatory T cells (Tregs) are particularly abundant and potent during pregnancy and at birth and inhibit excessive immune responses whereby ultimately the maintenance of peripheral T-cell tolerance and pathogen clearance are key [1,2,3,4]. Moreover the signaling within the intestine through pathogen recognition receptors like the toll like receptor (TLR) family for instance is crucial for the generation of effective immunity. Illustrative for this is that TLR2 influences the function of Tregs [5] and establishes a direct link between the intestinal microbiota and the control of immune responses through Tregs [6]. Tregs are important cells involved in immune regulation and play an increasing role in many immune related disorders of which many are found to be related to disturbed Treg function. For instance in HIV disease progression, the up regulation of fork head box p3 (Foxp3) expression in CD4^+^ T cells seems to be a marker of disease severity [7] and also in HAART-therapy treatment the Treg activity is associated with persistently reduced CD4^+^ T-cell counts during antiretroviral therapy [8]. Even, in chronic diseases like asthma, allergy, cancer an altered Treg number or function has been described. This is exemplified by the increased number of Tregs in children with eosinophilic esophagitis and explicit role of Tregs in tumor immunity [9]. In addition, related towards acute infections and innate immunity, an important role for Tregs is the suppression of innate immune pathology during influenza A virus infection [10]. Foxp3 ^+^ CD4^+^ Tregs limit pulmonary immune pathology by modulating the CD8^+^ T cell responses during respiratory syncytial virus infection [11,12]. These findings contribute to the notification, that next to immune response induction, a regulated suppression is essential for maintaining proper immune balance.

The diversity in immune responses evoked upon pathogen recognition may require several subsets of CD4^+^Foxp3^+^ Tregs to maintain proper immune homeostasis. There have been important functions reported for T-bet (Th1-specific T box transcription factor) and IRF-4 (Interferon regulatory factor 4) in Tregs demonstrating that the suppression ability requires the expression of transcription factors typically associated with the effector T cell function at place [13]. IRF4 is a decisive factor during Th17 development by influencing the balance of Foxp3, retinoid-related orphan receptor (RORα), and RORγt [14]. In response to IFN-γ, the Tregs are found to up regulate T-bet which promotes the expression chemokine receptor CXCR3, and T-bet^+^ Tregs accumulate at sites of Th1 cell–mediated inflammation. T-bet expression is required for the homeostasis and function of Treg cells during type 1 inflammation [15]. This subscribes the hypothesis that specific subsets of Foxp3^+^ Tregs develop for the suppression of Th1 responses *in vivo* [13]. Moreover, besides strong stimulation of effector T cells a modulation of Tregs to control immune responses provide an additional arm of intervention in the development of improved vaccines.

Early in life establishment of immune responsiveness is influenced by several factors, including nutrition. Breast milk contains several interesting immune modulating components with specific modulating potentials, which are known to have a clear role in immune mediated disease resistance later in life [16]. Specific oligosaccharides are known to modulate immune responses, as they can improve the immune balance in infants, resulting in lower incidence of infections and simultaneously can have an impact on allergy related symptoms [17]. Prebiotic oligosaccharides can have a direct effect via activation or inhibition of cellular receptors on immune competent cells [18] and may act indirectly through microbiota-dependent mechanisms (i.e. rebalancing microbiota composition in the gut) [19] The pre- and probiotic concept is based on the fact that our microbiota are considered to contribute to induction and maintenance of immune homeostasis possibly via CD25^+^ Tregs [20]. More specifically, it was found recently that Tregs play a fundamental role in the immune modulation induced by the supplementation of these specific oligosaccharides [21]. The exact underlying mechanism by which prebiotic oligosaccharides induce immune modulation effects however remains to be elucidated and is subject of current investigation. Given the importance of Tregs in maintenance of immune homeostasis and vaccine efficiency in the host, the specific role of Tregs in the immune-modulating effects of dietary supplementation with a unique mixture of prebiotic oligosaccharides scGOS/lcFOS/pAOS in a vaccination model has been investigated.

## Materials and Methods

### Animals and diets

Eight-week-old old male specific pathogen-free inbred C57BL/6J mice and C57BL/6-Foxp3tm1Flv/J were obtained from Charles River (Someren, the Netherlands) and housed under standard housing conditions with a 12 hr. dark and light cycle. All animals had free access to tap water and the semi-purified AIN-93G diet (Research Diet Services, Wijk bij Duurstede, the Netherlands), with or without oligosaccharide mixture consisting of three different prebiotic oligosaccharide materials, i.e. short-chain galacto-oligosaccharides (scGOS: Borculo Domo, Zwolle, 45% scGOS), long-chain fructo-oligosaccharides (lcFOS: Orafti, Wijchen, 100% lcFOS) and pectin hydrolysate derived acidic-oligosaccharides (pAOS: Sudzucker, Mannheim, 85% galacturonic acid). The oligosaccharides were mixed in a ratio of 9:1:10 based on carbohydrate purity. Although other combinations of these specific prebiotic oligosaccharides are known to be effective as well, it was this specific ratio within currently used mouse vaccination model which gives the largest immune modulation, detected by a DTH increase at the time and was therefore used for these mechanistically studies. A small negative control group of animals (n=3) was included only to show specificity of the vaccination procedure. Therefore, this group was not used for any statistical comparison to supplemented groups (n=10 per group). Fourteen days prior to the first vaccination dietary supplementation started which was maintained during the entire experimental procedure. The study protocol was reviewed and approved by the Animal Experimental Committee of the Utrecht University (permit number 2011.II.06.102).

### Vaccination protocol

All mice except the small negative control group received primary vaccination (day 1) and a booster vaccination (day 21) with a human influenza subunit vaccine consisting of haemagglutinin proteins of 3 different influenza strains (Influvac^®^, Solvay Pharmaceuticals, Weesp, the Netherlands). Vaccinations were performed by subcutaneous injection of vaccine (30 μg/ml per subunit) in a total volume of 100 µL. The negative control group received concurrent injections with PBS in a total volume of 100 µL. Vaccine-specific DTH reactions were induced 7 days after the last vaccination, by subcutaneous injection of 25 μL Influvac (30 µg/mL per haemagglutinin subunit) into the ear pinnea of one ear. For control, the other ear was injected with 25 μL PBS. Ear thickness was measured in duplicate before challenge, and 24 hours thereafter, with a digital micrometer (Mitutoyo Digimatic 293561, Veenendaal, the Netherlands). The influvac specificity of the DTH response was calculated by subtracting the basal ear thickness from the value at 24 hours after challenge and was corrected for the control swelling.

### Flowcytometric analysis

Splenocytes and cells from the mesenteric lymph nodes (MLNs) were isolated by gently pressing the organs through nylon mesh filters (Falcon cell strainer, Becton Dickinson, Alphen a/d Rijn, the Netherlands). After erythrocyte lysis (spleens only), a total of 1x10^6^ cells were washed with PBS containing 1% FBS and incubated for 15 min with an anti-mouse CD16/CD32 antibody (BD Pharmingen cat# 553142) blocking the Fc receptors. The cells were then stained with different combinations of anti-CD4-FITC (BD Pharmingen cat# 553046), anti-CD4-PE (BD Pharmingen cat# 553049), CD4-PeCy5 (BD Pharmingen cat# 553654), anti-CD25-PE (Beckman Coulter 732091), anti-CD69-APC (eBioscience cat# 17-0691), anti-CXCR3-APC (eBioscience cat# 17-1831), anti-CD103-PE (eBioscience cat# 12-1031), anti GITR-PE (eBioscience cat# 12-5874), or with anti CTLA-4-PE (eBioscience cat#12-1522) for 30 minutes. Intracellular stainings were performed according manufacturer’s protocol, (EBioscience, Foxp3 staining set, Bio connect, The Netherlands). For intracellular staining the antibodies anti-Foxp3-FITC (eBioscience cat# 11-5773), anti-Gata-3-PE (eBioscience cat# 12-9966), anti-T-bet-PerCP-Cy5 (eBiosience cat# 45-5825) were used in combination with above mentioned surface markers. Matching Isotype controls were used for all staining to minimize the influence of nonspecific binding, and proper gate setting. All staining procedures were performed on ice and protected from light. In total a minimum of 50.000 cells were counted and analyses were performed using FACSCanto II and FACSDiva software (BD Biosciences).

### Suppression assay

After labeling of MLN and spleen single cell suspensions obtained from C57BL/6-Foxp3tm1Flv/J mice (Charles river) with CD4-FITC and CD25-APC the CD4^+^CD25^+^mRFP^+^ Tregs were isolated by flow cytometry using a FACS Aria cell sorter. Sorted Tregs were cultured together with 2 µM pacific blue succinimidyl ester (PBSE Invitrogen) labeled total spleen cells (20.000 per well (

(Teff)) in ratio’s as indicated in the graphs. Cells were stimulated with 1ug/ml anti-CD3 (BD Pharmingen clone 145-2c11) for 96hr. at 37°C. Cell culture supernatants were collected and cytokines were measured according to manufacturer’s protocol using Bio-Plex Pro Mouse Cytokine Grp I panel 23-Plex (BioRad). For cell division analysis using PBSE positivity, the cells were stained with CD4-FITC (BD Pharmingen cat# 553046), CD8-APC (BD Pharmingen cat# 553932) and cell division was measured using flowcytometry.

### Statistical analysis

All statistical calculations were performed using SPSS version 12.0.1 software and Graph Path prism 4.03 software. Statistical differences between test and control groups were analyzed by one way ANOVA followed by multi-group comparison analysis using the Bonferroni test; otherwise an un-paired two sided t-test was used. Correlations were identified using Spearman and Pearson tests. All values are presented as mean ± SEM. P-values 0.05 were considered significant.

## Results

### Oligosaccharide induced T-bet / Gata-3 differentiation increase the Th1 responsiveness

**Figure 1:**
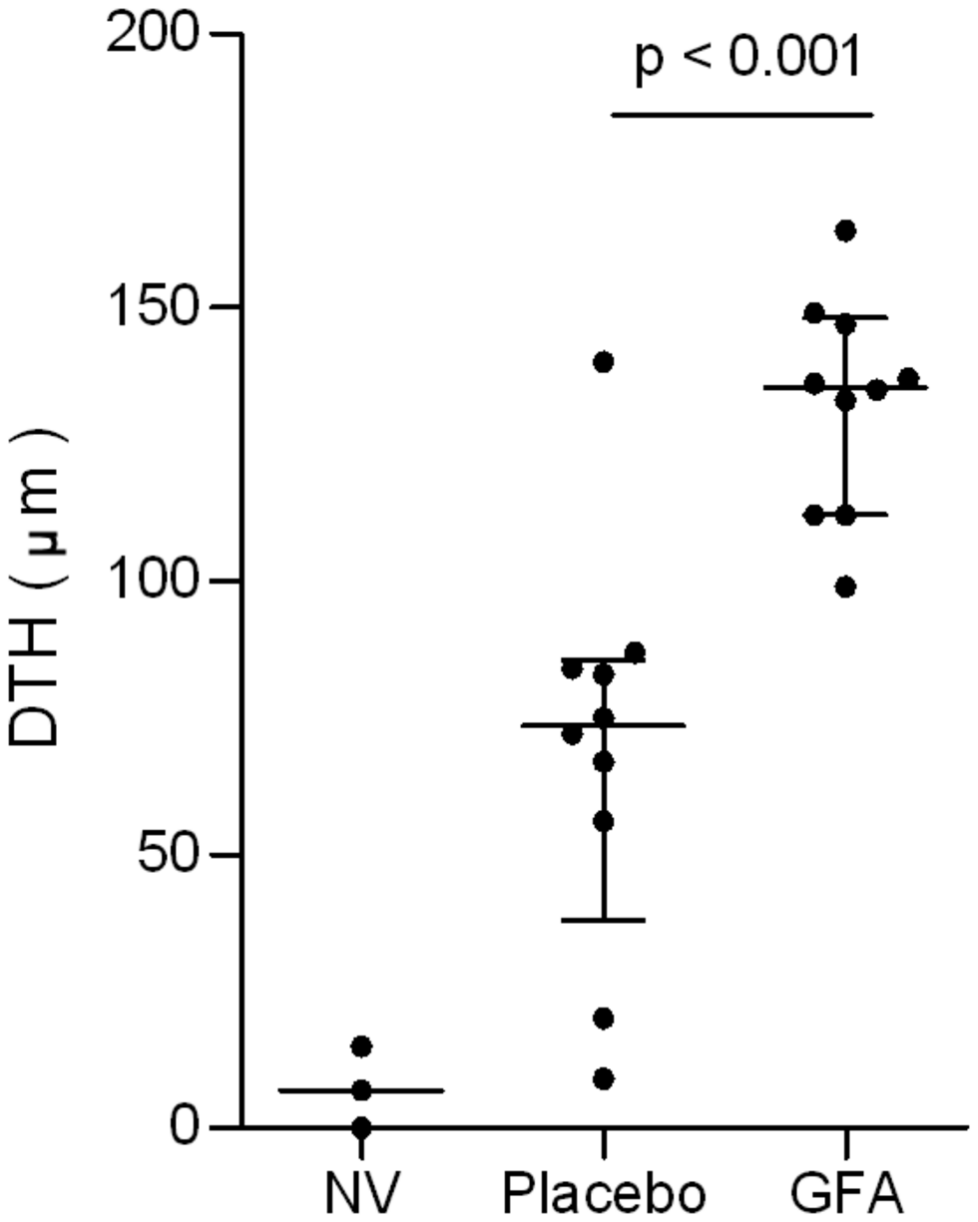
Dietary intervention alters Flu-specific DTH responses. C57Bl/6J mice (n = 10 per group) received a sub-maximal vaccination and dietary intervention with or without scGOS/lcFOS/pAOS during the entire vaccination procedure. Antigen specific DTH responses were measured by ear swelling (24 hr. post-antigen injection) and corrected for background (PBS) swelling. A non-vaccinated group (n = 3) was used as control (NV). Lines represent median with interquartile range of the DTH responses from individual mice per group (as indicated through separate dots). The statistical differences are indicated in the graph.

**Figure 2:**
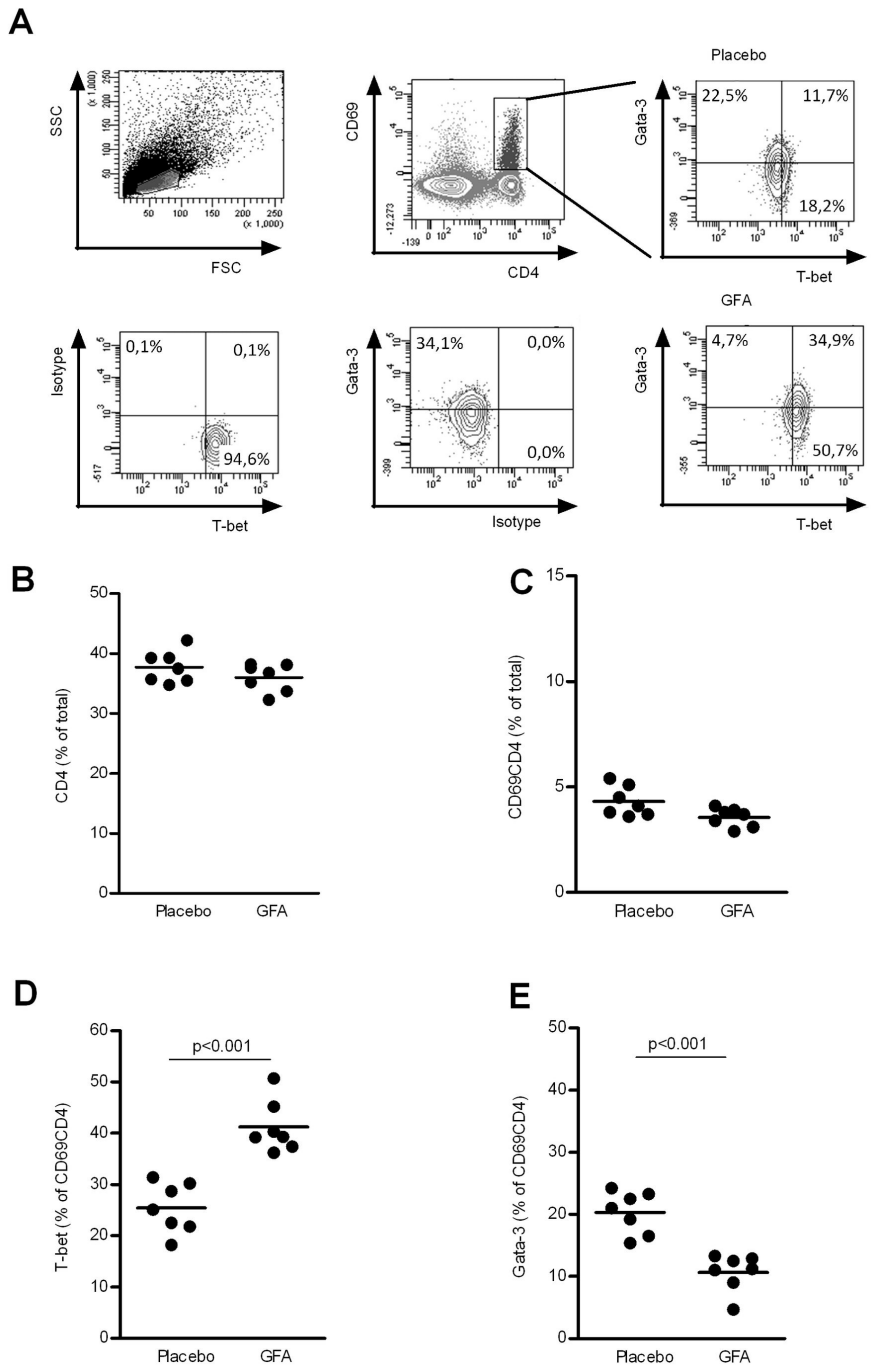
Activated CD4^+^ T-cells are modulated towards Th1 type of immune responsiveness. MLN cells were isolated from mice (n = 7 per group) receiving either placebo or scGOS/lcFOS/pAOS and were labeled with CD4/CD69/T-bet/Gata-3 flowcytometric analysis. The characterization of different cell populations is indicated in the gating strategy (A). Lines represent mean CD4^+^ T cells % (B), activated CD69^+^CD4^+^ T cells % (C) % of T-bet^+^ activated T-cells (D) and % of Gata-3^+^ activated T cells (E). In addition, individual measurements are indicated through separate dots. Data presented is representative for 3 individual experiments. Statistically significant differences between the groups are indicated in the graphs.

**Figure 3:**
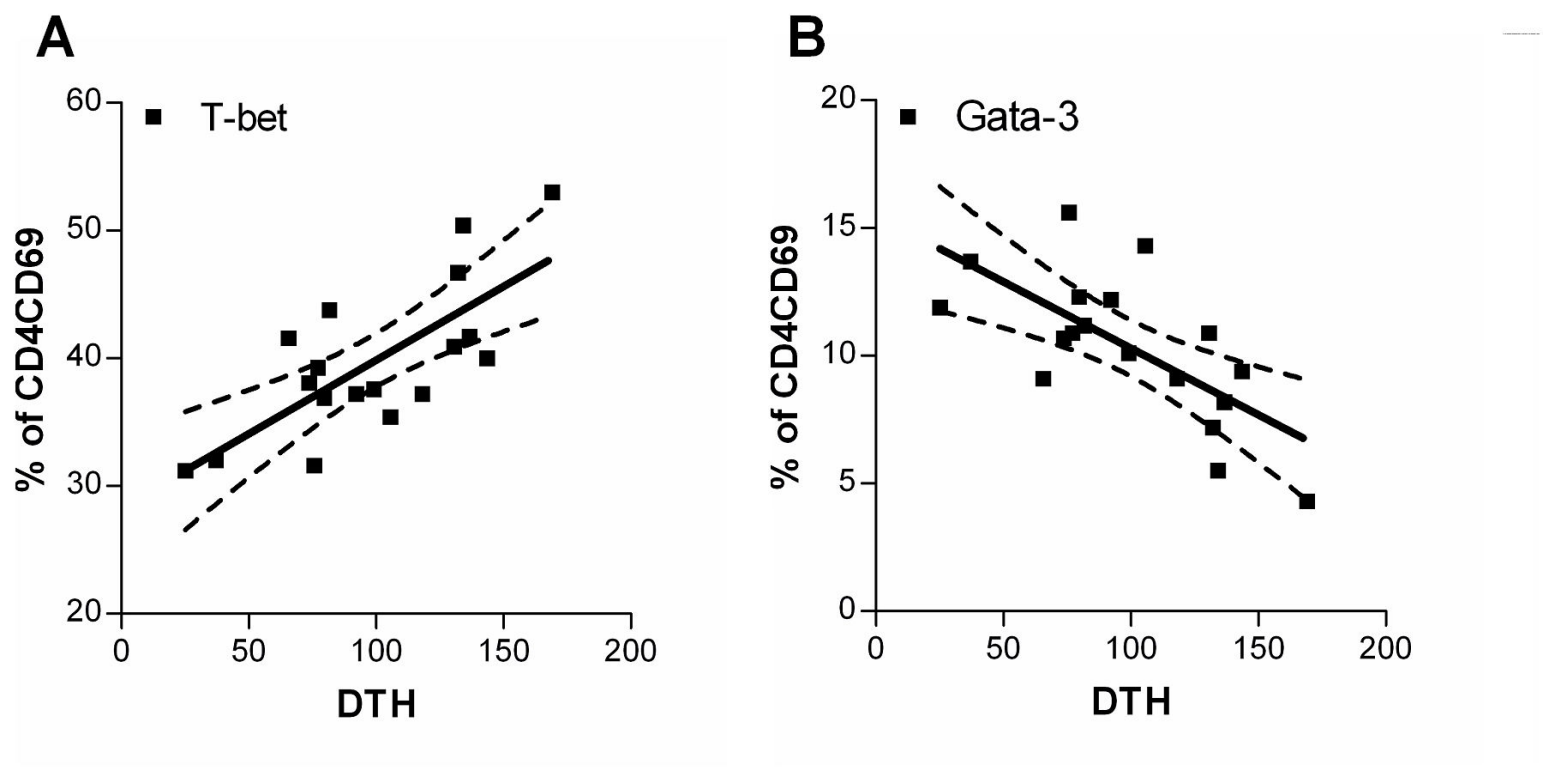
Correlation between DTH response and percentage of T-bet^+^, Gata-3^+^ of activated CD4 T cells in MLN. From individual mice the DTH values were correlated to the percentage of T-bet ^+^ (A) or percentage Gata-3^+^ (B) of the activated CD4^+^ T cells irrespective of dietary intervention, using Pearson and Spearman correlation tests. Mean correlation (line) and 95% CI (dashed) are indicated in the graphs next to the individual data points. With r2 = 0.5438 and r2 = 0.4653 for T-bet and Gata-3 respectively these percentages of activated T cells correlate significantly p0.001 and p0.01 respectively.

Immune modulation effects of the dietary intervention were analyzed by measuring antigen specific DTH responses, representing an *in vivo* parameter for Th1 type of cellular immunity. In mice receiving scGOS/lcFOS/pAOS, influenza vaccination results significantly (p0.001) increased influenza-specific DTH responses compared to mice receiving placebo diets (Figure 1) which are comparable with earlier described immune modulation capacities of orally supplied oligosaccharides [22]. In addition, the expression of T-bet and Gata-3 in the activated (CD69^+^) CD4^+^ T-cell population was assessed both in spleen (data not shown) and MLN cells (Figure 2). “Neither in the total CD4^+^ T cell (Figure 2B), nor in the percentage of activated CD69^+^CD4^+^ T cell populations (Figure 2C) is a significant change seen between the dietary interventions Interestingly a statistical significant increase in percentage of T-bet positive CD4^+^CD69^+^ T cells (*p0.001*) could be detected in MLNs of mice receiving scGOS/lcFOS/pAOS compared to mice receiving placebo diet (25.41 ± 1.85 placebo vs 41.19 ± 1.92 scGOS/lcFOS/pAOS % of CD4^+^CD69^+^ T cells (mean +/- SE)) as depicted in Figure 2D. In addition, a statistical significant reduction (p0.001) was found in the percentage of Gata-3 expressing activated CD69^+^CD4^+^ T cells (20.30 ± 1.28 placebo vs 10.66 ± 1.13 scGOS/lcFOS/pAOS % (mean +/- SE)) as depicted in Figure 2E. Moreover, a significant (p0.001) positive linear correlation (r^2^ = 0,54) was detected between the percentage of activated T-bet^+^ T cells and *in vivo* DTH response as well as a significant (p0.01) negative correlation (r^2^ = 0,47) between the percentage of activated Gata-3^+^ T cells and DTH response (Figure 3A,B). These data combined are indicative for improved Th1 type of immune responsiveness in C57BL/6J mice towards a fixed antigen dose due to specific dietary oligosaccharides.

### Changes within Treg population are in line with increased Th1 responsiveness

**Figure 4:**
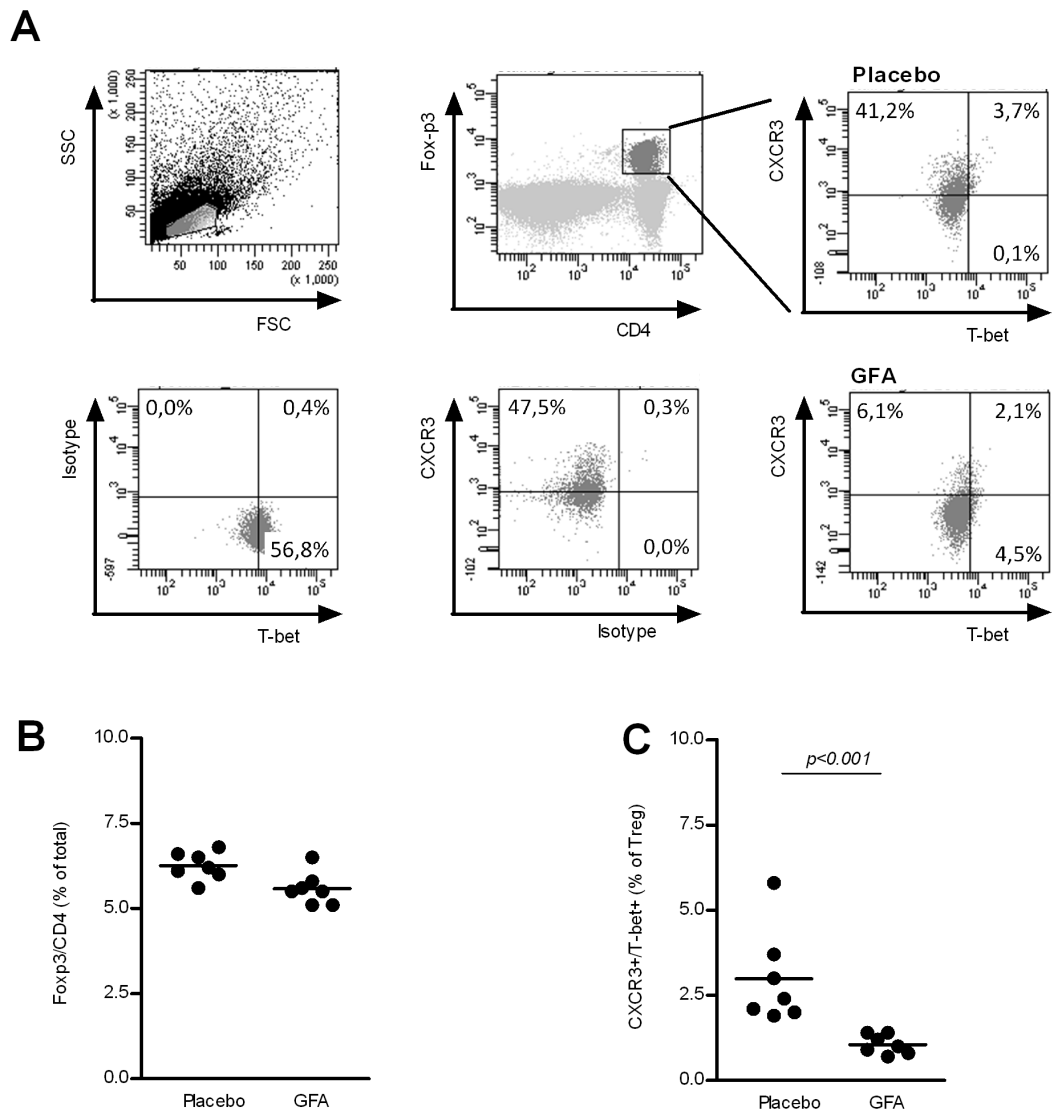
Treg population in MLN is influenced by the dietary intervention. Cells from MLNs were isolated from mice (n = 7 per group) receiving either placebo or scGOS/lcFOS/pAOS and were labeled with CD4/Foxp3 in combination with CXCR3, T-bet, for flowcytometric analysis (A). Lines represent mean percentages of Tregs in total (CD4^+^Foxp3^+^ T cells) (B), and sub-populations of Tregs including % of CXCR3 ^+^ /T-bet^+^ Tregs (C) In addition, individual measurements are indicated through separate dots. Data presented is representative for 3 individual experiments. Statistically significant differences between the groups are indicated in the graphs.

If the development of Tregs are in line with the developed immune response as postulated by Barnes et al. [13], than alterations in immune response should be accompanied with changes in Treg population accordingly. In order to test this hypothesis for the scGOS/lcFOS/pAOS induced Th1 responsiveness, the Treg population was analyzed using flowcytometry with surface staining of CXCR3 as additional marker besides T-bet expression (Th1 polarization) (Figure 4A). In mice receiving scGOS/lcFOS/pAOS the percentage of CD4^+^Foxp3^+^ T cells did not change in the MLNs (Figure 4B) or in the spleen (data not shown). Strikingly however, the percentage of Tregs were significantly lower (p0.05) in the MLNs of mice receiving scGOS/lcFOS/pAOS compared to mice receiving placebo diets (2.99 ± 0.53 placebo vs 1.06 ± 0.11 scGOS/lcFOS/pAOS % of Tregs (mean +/- SE)) (Figure 4C). Tregs (Foxp3 ^+^ CD4^+^) cells which are positive for both CXCR3 and T-bet are hypothesized to down regulate an increased Th1 response. Therefore these data are indicative for a reduced Th1 suppressive capacity in the Treg population of mice fed the scGOS/lcFOS/pAOS diet. Although the changed percentages are small some strongly significant immune modulatory changes in Treg population are detected due to specific oligosaccharides in the diet, showing their functional capacity as evidenced by increased DTH responses.

**Figure 5:**
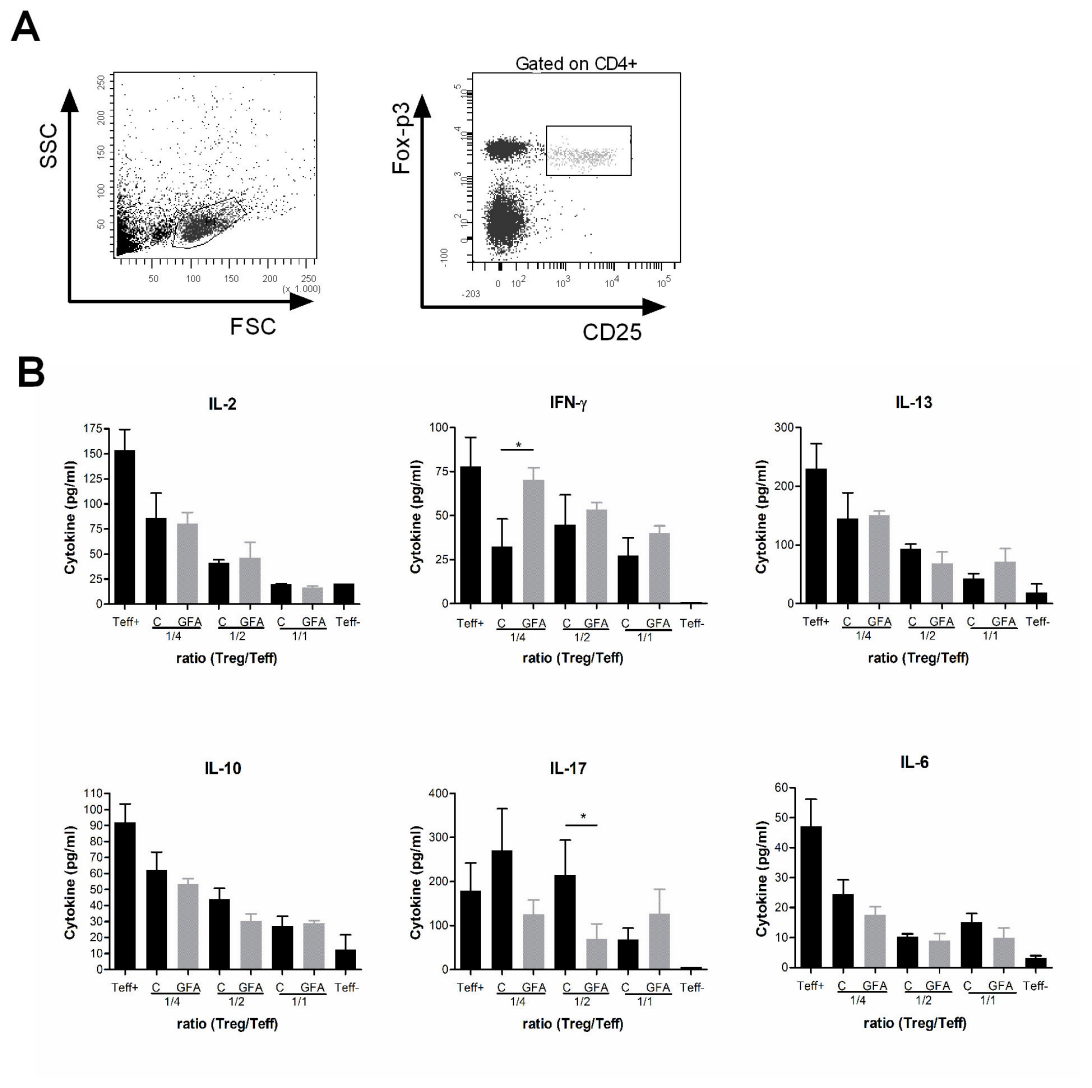
Suppression method but not capacity of Tregs is influenced by dietary intervention. CD4^+^CD25^+^mRFP^+^ regulatory T cells were isolated from MLN of the C57BL/6-Foxp3tm1Flv/J mice (n=4) using FACS Aria cell sorter and cultured with Teff cells in ratio of 1/1, 1/2, 1/4 (Treg /Teff) for 96hr. after stimulation with α-CD3. Cell culture supernatant contained different cytokines (B) including (IL-2, IFN- γ, IL-13, IL-10 and IL-17A). Only the significant differences between the diets were indicated using * for p0.05.

Finally the suppressive capacity of isolated Tregs from spleen as well as MLNs was analyzed. From C57BL/6-Foxp3tm1Flv/J mice the regulatory T cells (characterized as CD4^+^CD25^+^mRFP^+^) were cell sorted and cultured together with PBSE labeled effector T cells for 96 hours. Cell division was observed after CD3 stimulation for 96h. Although Tregs isolated from both the spleen as well as the MLN suppressed the CD3 induced T cell proliferation, in none of the ratios tested a difference could be observed between the Tregs isolated from the mice on the different diets (data not shown). In addition to an overall reduction in cytokines produced with increasing percentage of Tregs, a small change in some cytokines was detected between the diets. As shown in Figure 5 no difference in IL-2 (proliferation) could be detected, but a significant (p0.05) increased IFN- γ (Th-1) as well as significant (p0.05) reduced IL-17 (Th-17) response was detected in spleen cell populations suppressed by Tregs from mice receiving the specific oligosaccharides. The production of IL-13 (Th2), IL-10 as well as IL-6 (inflammatory), were suppressed with increasing amounts of Tregs, but not different between the diets. These changes are in line with improved Th1 type of immune responsiveness in C57BL/6J mice towards a fixed antigen dose due to specific dietary oligosaccharides.

## Discussion

A better understanding of the mechanism by which vaccine induced immune responses can be increased using alternative strategies will improve vaccine development. In our current study we show that specific modulation of the immune response by specific oligosaccharide containing diet results in alteration in Treg population. More specifically, a reduced percentage of T-bet^+^ Tregs were induced in mice fed scGOS/lcFOS/pAOS diet during vaccination, resulting in increased vaccine responsiveness. Although the involvement of Tregs in dietary immune modulations has been indicated before [21,23], this is the first study showing that an alteration of activated CD4^+^ T cells (increased T-bet (Th1) and reduced Gata-3 (Th2)) is accompanied with a reduced population Tregs expressing T-bet. Moreover, although suppressive capacity does not seem to be altered, the Tregs seem to suppress through a different and to hereunto unknown mechanism.

Recent observations have challenged the notification of one stable Treg sub lineage. Some reports suggest that there are multiple, functional Treg subsets. One of these subsets expresses T-bet, and has shown to be specifically adapted for the suppression of Th1 responses [15]. In addition another Treg subset expressing IRF-4 was identified, which is essential for Th2 differentiation. The absence of IRF-4^+^ Tregs even resulted in spontaneous Th2 mediated inflammation, suggesting the necessity of the IRF-4 positive Tregs to control Th2 type of responses [14]. Furthermore a third Treg subtype expressing STAT-3 seemed required suppressing Th17 responses [24]. Therefore as the plasticity of T cell population seems to require plasticity of Tregs to control excessive immune responses, this provides an additional arm of intervention. Within our studies we clearly show increased vaccination responsiveness, by dietary intervention. Both the increased DTH response as well as increased percentage of T-bet expressing T-cells is indicative for improved Th1 responsiveness, which is accompanied by alterations within the Treg population.

Previously, we observed a systemic decrease in Treg numbers in lung, spleen, and mesenteric lymph nodes at day 4 after infection in FI-RSV-vaccinated mice receiving the scGOS/lcFOS/pAOS diet. Tregs have previously been found to regulate RSV-specific primary immune responses. Within this study the expression of granzyme B (GzmB) in Treg locally in the lung was shown to be involved in the immune-regulatory function of Tregs in the primary RSV infection model [25]. Although the percentage of Tregs did not differ, a significantly decreased absolute number of Tregs was detected in the BAL fluid at day 4 after challenge in FI-RSV-vaccinated mice receiving the scGOS/lcFOS/pAOS diet. This indicates that the dietary intervention with scGOS/lcFOS/pAOS has an effect on the function of regulatory immune cells which correlates with altered immune responses.

The changes detected in the Treg population are multiple, they include a reduced percentage of CXCR3 ^+^ /T-bet^+^ Tregs, GITR ^+^ CXCR3^+^ Tregs (data not shown) in the MLNs of mice receiving scGOS/lcFOS/pAOS diet compared to mice fed control diet. T-bet controls migration to inflammatory sites through CXCR3 up regulation as CXCR-3 is a direct transcript of T-bet [26]. Thus, T-bet may therefore play an important role in the direction and regulation of regulatory T cells. This indicates that it is not the percentage of Tregs in total, but merely the functionality which changes due to dietary intervention. Tregs also express receptors for inflammatory chemokines (CCR4, CCR9, and CXCR3), integrin’s, and tissue-homing receptors like CD103 [27]. Indeed, it was demonstrated that CD103^+^ Tregs are attracted and retained in inflamed tissues where they may exert their suppressive function [28]. Functional compartmentalization of Tregs is linked to the expression of different phenotypes, with Tregs found in tissues expressing CD103, IL-10, IL-2R, and CCR5.

In conclusion; this study shows that with dietary intervention using specific oligosaccharides improved vaccine responsiveness can be induced due to reduced Th1 suppressive capacity in the Treg population of mice. The better understanding of the mechanism by which vaccine induced immune responses can be amplified using alternative strategies will improve vaccine development.
